# Consenso multidisciplinar para el seguimiento y control del asma mediante la telemedicina. El proyecto COMETA

**DOI:** 10.1016/j.opresp.2021.100098

**Published:** 2021-04-19

**Authors:** Carlos Almonacid Sánchez, Marina Blanco Aparicio, Javier Domínguez Ortega, Jordi Giner Donaire, Jesús Molina Paris, Navidad Sánchez Marcos, Vicente Plaza

**Affiliations:** a Servicio de Neumología, Hospital Universitario Ramón y Cajal, IRYCIS, Madrid, España; b Servicio de Neumología, Hospital Universitario A Coruña, A Coruña, España; c Servicio de Alergia, Instituto de Investigación del Hospital Universitario La Paz (IdiPAZ), Madrid, España; d Servicio de Neumología y Alergia, Hospital de la Santa Creu i Sant Pau, Barcelona, España; e Centro de Salud Francia, Dirección Asistencial Oeste, Fuenlabrada, Madrid, España; f Farmacia comunitaria, San Sebastián de los Reyes, Madrid, España; g Instituto de Investigación Biomédica Sant Pau (IIB Sant Pau), Barcelona, España; h Departamento de Medicina, Facultad de Medicina, Universidad Autónoma de Barcelona, Barcelona, España; i CIBER de Enfermedades Respiratorias (CIBERES), Madrid, España

**Keywords:** Telemedicina, Asma, Control, Protocolo, Telemedicine, Asthma, Control, Protocol

## Abstract

A pesar de los avances terapéuticos disponibles actualmente, el grado de control del asma es escaso. Dicho control se basa en evaluar al paciente, ajustar el tratamiento y revisar la respuesta al mismo. En situaciones normales, el seguimiento y el control del asma se realizan mediante visitas presenciales secuenciales. Sin embargo, debido a las medidas de bioseguridad y distanciamiento para evitar la transmisión de la enfermedad durante una pandemia, ese seguimiento y control se ven limitados. Así es como ha surgido la teleasistencia, la cual dispone de una amplia evidencia publicada en asma. Aun así, no ha de entenderse como una forma de sustituir a las consultas presenciales, sino como una alternativa complementaria a las mismas, en las que se permite el seguimiento de los pacientes cuando no sea necesario o no se pueda realizar una consulta presencial. A través del proyecto COntrol como Meta en la Era de la Telemedicina en el Asma (COMETA), un grupo de expertos abordó en profundidad la enfermedad asmática, analizando de forma detallada los problemas existentes para poder alcanzar el control, y proponer soluciones ante situaciones como las que estamos viviendo actualmente con la pandemia de la COVID-19.

## Abstract (en)

Despite the therapeutic advances currently available, asthma control is poor. Such control is based on assessing the patient, adjusting treatment, and reviewing the response to treatment. In normal situations, asthma is monitored and controlled by sequential face-to-face visits. However, due to biosecurity and distancing measures to avoid disease transmission during a pandemic, such monitoring and control is limited. This is how tele-assistance, which is available from extensive published evidence in asthma, has emerged. Even so, it should not be understood as a substitute for face-to-face consultations, but as a complementary alternative to them, in which patients can be monitored when a face-to-face consultation is not necessary or cannot be carried out. Through the COMETA project (*COntrol como Meta en la Era de la Telemedicina en el Asma*, Control as a Goal in the Age of Telemedicine in Asthma), a group of experts addressed in depth the asthmatic pathology, analyzing in detail the existing problems in order to achieve control and propose solutions to situations such as those we are currently experiencing with the COVID-19 pandemic.

## Introducción

La prevalencia del asma en España se estima en un 5% en adultos y un 10% en la población infantil^1^. Sin embargo, a pesar de la existencia de tratamientos farmacológicos cada vez más eficaces y seguros, tal como han reflejado los estudios INSPIRE, MAGIC y REALISE el grado de control del asma es escaso, con unas tasas de buen control que oscilan entre el 13 y el 28% desde 2006 a 2014^2^, ^3^, ^4^.

El control del asma se basa en 3 pilares fundamentales interconectados entre sí: evaluar al paciente, ajustar el tratamiento y revisar la respuesta al mismo^5^. Durante situaciones como la provocada por el confinamiento debido a la pandemia de la COVID-19, este control puede ser todo un desafío. En situaciones normales, la mayoría de los profesionales sanitarios realizan el seguimiento y el control del asma mediante visitas presenciales secuenciales. Sin embargo, debido a las medidas de bioseguridad y distanciamiento para evitar la transmisión de la enfermedad durante la pandemia^6^, se ha tenido que recurrir a otras alternativas que permiten hacerlo a distancia o de forma remota.

La prueba de elección para evaluar la función pulmonar en enfermedades respiratorias crónicas, como el asma, es la espirometría. Dada la situación actual, las versiones más recientes de la Guía Española para el Manejo del Asma (GEMA 5.0) y la Iniciativa Global para el Asma (GINA 2020) recomiendan garantizar las medidas de seguridad oportunas antes de llevarla a cabo^5^, ^7^. Por este motivo, muchos centros dejaron de practicarla durante los brotes más graves de la pandemia. De forma paralela, incluso se ha planteado la medición del flujo espiratorio máximo (FEM) mediante el uso del medidor de FEM como alternativa sencilla y de acceso fácil a cualquier nivel asistencial para evaluar el control del asma, especialmente a nivel domiciliario. Otras herramientas que han demostrado ser útiles en el seguimiento de los pacientes asmáticos, tanto de forma presencial como a distancia, son el test de control del asma (ACT)^3^, ^4^, ^7^ y el test de adhesión a los inhaladores (TAI)^8^. Este último incluso se debería complementar con el registro electrónico de retirada de fármacos en la farmacia^9^.

Durante el seguimiento de los pacientes asmáticos, los profesionales sanitarios médicos deben evaluar y ajustar el tratamiento, si fuera necesario. Las 6 acciones básicas a realizar en cada visita para alcanzar y mantener el control del asma, sea de forma presencial o a distancia, son: 1) determinar el control de los síntomas (mediante un interrogatorio estructurado o el ACT)^3^, ^4^, ^5^, ^7^; 2) realizar una medida objetiva de la función pulmonar (preferiblemente con una espirometría)^5^, ^7^; 3) evaluar la adhesión terapéutica mediante un método validado (a través del TAI y el registro electrónico de retirada de fármacos en la farmacia)^8^, ^9^; 4) revisar periódicamente que la técnica de inhalación sea la correcta, así como la satisfacción del paciente por el tratamiento^7^, ^10^; 5) ajustar el tratamiento de mantenimiento del control^5^, ^7^, y 6) realizar un seguimiento periódico del paciente a través de visitas presenciales o teleasistencia médica^7^. Este último punto ha supuesto todo un desafío desde el inicio de la pandemia de la COVID-19, teniendo que adaptar todo el sistema a una nueva modalidad de consultas mediante el uso de nuevas tecnologías.

El objetivo de este artículo es dar a conocer un consenso realizado por un grupo multidisciplinar de expertos involucrados en la enfermedad asmática, con el fin de ofrecer recomendaciones que sirvan de ayuda al personal sanitario en el seguimiento telemático de sus pacientes para alcanzar y mantener el control del asma. Esto es especialmente relevante en la situación actual de adaptación a una nueva modalidad de consultas, donde la teleasistencia médica es una herramienta complementaria de gran utilidad para el seguimiento de los pacientes asmáticos.

## Metodología

A través de varias reuniones virtuales, el grupo multidisciplinar de expertos en neumología, alergología, medicina de familia, enfermería y farmacia comunitaria debatió sobre cómo alcanzar y/o mantener el control de la enfermedad asmática en situaciones en las que no es posible el seguimiento presencial de estos pacientes, tanto antes como durante y después de la pandemia de la COVID-19.

Inicialmente se identificaron aquellos problemas que existen o pueden existir a medio y largo plazo en cada uno de sus ámbitos. Después se plantearon iniciativas que, según su práctica clínica habitual, podrían servir para solucionar los mismos. Finalmente, se consensuaron métodos para atender a los pacientes asmáticos a través de la teleasistencia médica.

## Identificación y priorización de los problemas existentes en el control de la enfermedad asmática. Iniciativas para paliar estos problemas

Los principales problemas identificados por el grupo de expertos fueron: el déficit de información sobre la cronicidad del asma en la población general, el elevado infradiagnóstico de la enfermedad, el escaso conocimiento específico y técnico sobre su manejo, la falta de adhesión terapéutica por parte de los pacientes, los problemas derivados de técnicas de inhalación incorrectas, la limitada comunicación entre los profesionales sanitarios involucrados en la enfermedad, la falta de seguimiento periódico y la escasa concienciación sobre esta enfermedad entre los sanitarios y la población general pese a los esfuerzos empleados en promover la formación y la educación en el asma.

Las principales iniciativas propuestas se centraron en la concienciación de la población general sobre la importancia de identificar los síntomas respiratorios y, una vez diagnosticado, alcanzar y mantener el control del asma. Para ello, se destacó el papel importante que podría desempeñar la farmacia comunitaria, pues estos profesionales tienen un contacto directo en el día a día con los pacientes, especialmente en situaciones en las que el sistema sanitario se encuentra congestionado. Así mismo, también se consideró muy importante mejorar la comunicación entre los profesionales sanitarios. También se destacó el papel del personal de enfermería, dada su cercanía con los pacientes y su capacidad para poderles informar, formar y educar. Del mismo modo, se compartió el enorme papel que puede realizar en el día a día tanto enfermería como farmacia comunitaria en la detección de pacientes asmáticos y en el mal control de la enfermedad. Junto a todas estas propuestas, se planteó la posible utilidad de aplicaciones intuitivas y de acceso fácil para pacientes y profesionales sanitarios, así como la disponibilidad de un número de teléfono al que los pacientes puedan recurrir para consultar sus dudas e inquietudes.

## Teleasistencia de los pacientes asmáticos

Hasta la pandemia de la COVID-19, la implementación de la teleasistencia había sido fundamentalmente experimental, pero desde entonces se ha convertido en un procedimiento fundamental que ha demostrado ser útil para garantizar tanto el control de las enfermedades crónicas como el seguimiento de estos pacientes^11^.

La teleasistencia médica engloba la telemonitorización (encargada de recopilar datos del paciente), la teleconsulta (consulta remota entre paciente y clínico) y la telemedicina (consulta remota entre médicos para compartir experiencias y consultar casos clínicos)^7^. Todas ellas pueden realizarse mediante un ordenador, una tableta digital o incluso el teléfono, pero siempre condicionadas a las capacidades y limitaciones de cada centro sanitario y de acuerdo al marco legal establecido. Sus objetivos son mejorar la gestión individualizada y la calidad de vida de los pacientes, así como mejorar el control del asma.

La evidencia sobre la teleasistencia es muy amplia, con más de 3.000 artículos publicados, de los cuales más de 300 hacen referencia a la telemedicina y al asma^12^. En todos ellos se demuestra su eficacia para el control del asma y el seguimiento de los pacientes. Algunas publicaciones han abordado el uso de la teleasistencia de una forma muy general, como la guía de la Sociedad Española de Neumología y Cirugía Torácica (SEPAR) sobre el seguimiento telemático de pacientes con enfermedades y procesos respiratorios^13^. Otros proyectos que se están desarrollando actualmente, abordan de forma específica el manejo del asma grave mediante la telemedicina.

Los principales beneficios de la teleasistencia en la práctica clínica habitual son: 1) prevenir el riesgo de contagio en épocas de viriasis; 2) controlar la enfermedad de los pacientes; 3) optimizar los recursos sanitarios; 4) ahorrar visitas y desplazamiento de los pacientes; 5) facilitar el seguimiento continuado de los pacientes, y 6) permitir la colaboración entre profesionales sanitarios. El objetivo de este último punto es optimizar los recursos, participar en el seguimiento de los pacientes y detectar los pacientes mal controlados para su derivación.

La teleconsulta no ha de entenderse como una forma de sustituir a las consultas presenciales, sino como una alternativa complementaria a las mismas, en las que se permite el seguimiento de los pacientes cuando no sea necesario o no se pueda realizar una consulta presencial. Además, al igual que la telemedicina, está dirigida a facilitar la comunicación entre los profesionales sanitarios involucrados en el control y en el seguimiento de los pacientes.

Actualmente, en España no existe una normativa concreta sobre la teleasistencia, aunque algunas comunidades autónomas han propuesto diferentes iniciativas. Sin embargo, sí existen leyes a nivel europeo que abordan parte de ella^14^, ^15^. La teleasistencia ha de considerarse como un acto legal, siempre que cumpla con el Reglamento General de Protección de Datos (RGPD) y se tengan en cuenta las medidas excepcionales de la pandemia de la COVID-19^16^, ^17^. Pasada esta, tendrán que regularse todas las lagunas legales existentes. Actualmente no se requiere un consentimiento por escrito, pero según el RGPD, si la imagen permite identificar al paciente y se usa con fines docentes o científicos, este ha de ser informado y ofrecer su autorización^17^, ^18^. Por este motivo, es importante que el paciente reciba toda la información necesaria, conozca la dinámica del proceso y pueda ofrecer su consentimiento para poder respetar su autonomía.

Desde la Comisión Central de Deontología, Derecho Médico y Visado (CCD) de la Organización Médica Colegial de España (OMC) existe un posicionamiento claro sobre la teleasistencia, considerándola como un complemento de la atención presencial y que nunca ha de ser impuesta, sino consensuada por parte de los implicados^19^.

## Algoritmo de teleconsulta para pacientes asmáticos

El grupo de expertos del presente artículo, y a través del proyecto COntrol como Meta en la Era de la Telemedicina en el Asma (COMETA), abordó en profundidad la enfermedad asmática, analizando de forma detallada los problemas existentes para poder alcanzar el control y proponer soluciones ante situaciones como las que estamos viviendo actualmente con la pandemia de la COVID-19.

**Figura 1:**
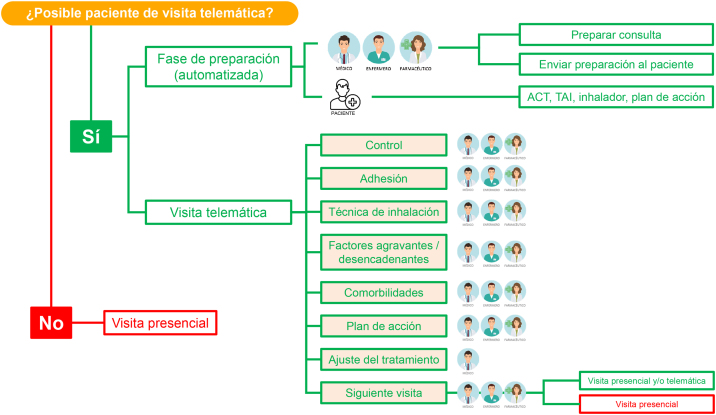
Algoritmo de actuación para el proyecto COMETA.

En la figura 1 se muestra un algoritmo de visita telemática a un paciente con asma y se detalla qué profesionales sanitarios podrían participar en cada una de sus fases. Antes de plantear esta visita, es necesario comprobar si se puede realizar de forma adecuada. Para ello, el paciente debe cumplir una serie de criterios: disponer de teléfono o Internet, no presentar una discapacidad que le impida usar estos sistemas y, sobre todo, querer emplearlos. Es importante tener en cuenta que el consentimiento del paciente para realizar una visita telemática se produce en el mismo momento que este desea llevarla a cabo, y así ha de quedar reflejado en su historia clínica.

En los pacientes que así lo decidan, la teleconsulta se plantea en 2 fases: una fase de preparación previa y la teleconsulta propiamente dicha. La fase de preparación se puede y debe automatizar mediante un sistema de gestión para que el paciente sepa que va a recibir una consulta telemática. Esto se realiza mediante el envío de un correo electrónico o un mensaje de texto en el móvil que avisen y recuerden al paciente que puede conectarse a través de una plataforma segura. También puede emplearse una aplicación móvil específica que englobe tanto las notificaciones como la conexión a la teleconsulta. Junto a la notificación, se enviará al paciente el ACT, el TAI de 10 ítems (TAI-10) y se le solicitará que tenga a mano su inhalador y el plan de acción, así como el medidor de FEM con su registro (quien disponga de él). Por parte de los profesionales sanitarios (médicos, enfermeros y farmacéuticos), se revisará la historia clínica del paciente y el registro electrónico de retirada de fármacos en la farmacia.

La segunda fase es la visita telemática propiamente dicha. Aunque existen varias alternativas, la forma más habitual y accesible a día de hoy para la mayoría de los pacientes y centros sanitarios, es la llamada telefónica. Se trata de una visita estructurada de la misma manera que una visita presencial. En ella se valorará y revisará el control del asma en el paciente, su adhesión al tratamiento, la técnica de inhalación, los factores agravantes y/o desencadenantes y la presencia de comorbilidades. Con todo ello, el médico ajustará el tratamiento, se modificará el plan de acción y se planificará la visita siguiente.

• *Control del asma:* para ello se revisa el ACT cumplimentado por el paciente, se pregunta por el número de agudizaciones desde la última visita, si ha tomado corticosteroides orales, el uso de medicación de rescate (sea un agonista β2 de acción corta [SABA] o larga [LABA]), la presencia de síntomas nocturnos o diurnos, y se revisa el registro del medidor del FEM (en aquellos pacientes que dispongan de él). Aunque el medidor del FEM presenta sus limitaciones, y ya existen espirómetros portátiles, se trata de una técnica que proporcionará información útil sobre el grado de obstrucción. El control del asma puede ser valorado por médicos, enfermeros y farmacéuticos. Sin embargo, en caso de mal control, el paciente debe ser valorado por su médico para evaluar y ajustar el tratamiento, así como revisar el plan de acción.
• *Adhesión terapéutica:* para ello se revisa el TAI-10 cumplimentado por el paciente en la fase de preparación y el registro electrónico de retirada de fármacos en la farmacia. Puede ser llevada a cabo por médicos, enfermeros y farmacéuticos.
• *Técnica de inhalación:* para su revisión es necesario disponer de una cámara o vídeo que permita visualizar el procedimiento llevado a cabo por el paciente y poder corregir los errores. En caso de que el paciente no disponga de cámara, o no sea posible visualizarlo a través del ordenador de la consulta, puede ofrecérsele vídeos con tutoriales sobre la técnica de inhalación. El principal problema de los videotutoriales es que no se puede objetivar si el paciente hace bien la técnica de inhalación o no. Ante esta situación, lo aconsejable es remitir al paciente al personal de enfermería o farmacia comunitaria. De hecho, estos últimos, por su cercanía con los pacientes, son los que más ayuda pueden proporcionarles cuando van a recoger su dispositivo inhalador a la farmacia.
• *Factores agravantes y/o desencadenantes:* durante la visita telemática también se han de revisar los consejos de autocuidado del asma, identificando los factores agravantes y/o desencadenantes de síntomas y exacerbaciones, y recordar evitarlos, y si el asma del paciente tiene o no relación con algún componente laboral o incluso relacionado con la ansiedad. Todo ello puede ser llevado a cabo por médicos, enfermeros y farmacéuticos.
• *Comorbilidades:* el objetivo es identificar si el paciente padece alguna comorbilidad que pudiese contribuir a un mal control o incrementar el riesgo de exacerbaciones, como obesidad, síndrome de apnea-hipopnea del sueño, rinosinusitis crónica, reflujo gastroesofágico, tabaquismo, alergia alimentaria o embarazo. Una vez identificadas las comorbilidades, se proponen las oportunas medidas terapéuticas. Esta tarea puede llevarla a cabo inicialmente el personal de enfermería y, posteriormente, los médicos. Desde farmacia comunitaria también puede hacerse un seguimiento de las comorbilidades, especialmente en situaciones en las que los pacientes solicitan medios para tratar algunas de ellas, como los reflujos, o cuando el farmacéutico detecta un inusual aumento de peso, consumo de tabaco y/o derivados, o una disminución de adhesión terapéutica por miedo a seguir el tratamiento durante el embarazo.
• *Plan de acción:* del mismo modo que durante una visita presencial convencional, se ha de revisar y ajustar el plan de acción junto con el paciente, ofreciendo pautas a seguir según la situación clínica de su asma. Aunque el plan de acción es elaborado por el médico, el personal de enfermería y farmacia comunitaria puede ayudar en su revisión, explicación y comprensión. Desde farmacia comunitaria también se pueden detectar aquellos pacientes sin un plan de acción definido o mal establecido, derivándoles a sus médicos para que se les implemente o revise^20^, ^21^.
• *Evaluación y ajuste del tratamiento:* cuando el paciente está bien controlado se podría considerar el descenso del tratamiento o, en caso de no poderse plantear, mantener el tratamiento sin cambios. En caso de mal control, mala adhesión, o cualquier otra circunstancia que requiriese la intervención del médico, es necesario comprobar y corregir la causa del mal control y reajustar el tratamiento.
• *Programación de la siguiente cita:* si el control hubiese sido satisfactorio y no fuera necesario el ajuste del tratamiento, la siguiente cita podría ser telemática y/o ser realizada por el personal de enfermería. Por el contrario, en caso de no haberse conseguido el control del asma, se plantearía una visita presencial. En ocasiones, se pueden evitar las visitas presenciales a la consulta médica gracias a las consultas de enfermería, las cuales pueden ayudar a controlar el asma mediante cuestionarios, formación y educación. Gracias a estas consultas telemáticas se optimizan los recursos sanitarios y se evita el desplazamiento de los pacientes a los centros de salud u hospitales. Aunque el personal de farmacia comunitaria no esté incluido en el circuito del Sistema Nacional de Salud mediante el acceso a la historia clínica, en ocasiones, y tras el consentimiento del paciente, puede ayudar a concertar una cita con el médico o el personal de enfermería si considera que el paciente no está bien controlado o necesita ayuda con los nuevos sistemas digitales de petición de cita.

## Proyecto COMETA

COMETA es un proyecto docente orientado a la gestión clínica que cuenta con la participación multidisciplinar de expertos en neumología, alergología, medicina de familia, enfermería y farmacia comunitaria. Su objetivo es formar a los profesionales sanitarios ante la necesidad de adaptarse a un entorno en el que las teleconsultas están cobrando cada vez más importancia, proporcionando herramientas útiles para la práctica clínica diaria que faciliten el seguimiento y el control del asma más allá de periodos de confinamiento por pandemias. Las bases de este proyecto basculan en 3 ejes fundamentales. El primero de ellos es el control del asma, y sobre él descansan los otros 2, la teleasistencia médica, como herramienta complementaria para facilitar el seguimiento de los pacientes, y la colaboración entre profesionales sanitarios, para mejorar la calidad asistencial. Con todo esto, COMETA nace como un proyecto de soporte y guía durante este tiempo de transformación hacia una nueva forma asistencial que combina la asistencia a las consultas de forma presencial con la teleasistencia, manteniendo siempre el foco en mejorar el control y calidad de vida en el asma.

COMETA está formado por un comité científico-académico independiente y cuenta con el soporte de GSK.

La web del proyecto COMETA es: https://gskpro.com/es-es/iniciar-sesion/iniciarsesion/?resource=%2Fes-es%2Fareas-terapeuticas%2Frespiratorio%2Fpatolog%25C3%25ADa-asma%2Fproyecto-cometa%2F

## Conclusiones

Aunque la teleasistencia sanitaria ya existía previamente, la pandemia provocada por la COVID-19 ha propiciado su implantación como un procedimiento fundamental para garantizar el control del asma más allá de cualquier situación restrictiva. La teleasistencia médica ha de verse como una herramienta complementaria a la práctica clínica habitual para reducir aquellas consultas presenciales no necesarias, y optimizar y aliviar al ya de por sí saturado sistema sanitario.

Por ello, COMETA intentará acompañar y servir de apoyo en este periodo de adaptación hacia una nueva medicina que aúna la consulta presencial y la teleasistencia médica, con el objetivo de lograr el control de la enfermedad asmática y con ello, mejorar la calidad de vida de sus pacientes. El paso siguiente del Comité Científico del proyecto COMETA es «validar» las recomendaciones ofrecidas en este artículo mediante un consenso Delphi en el que participe un grupo amplio de expertos.

## Financiación

El trabajo no ha recibido ayuda financiera.

## Conflicto de intereses

COMETA es un proyecto soportado por GSK que está constituido por un comité científico-académico independiente formado por los autores del manuscrito.

Carlos Almonacid Sánchez declara haber recibido honorarios por su participación en reuniones, asesorías, congresos o trabajos de investigación organizados por las siguientes industrias farmacéuticas: ALK, AstraZeneca, Chiesi, GSK y Novartis.

Marina Blanco Aparicio declara haber recibido honorarios por su participación en reuniones, asesorías, congresos o trabajos de investigación organizados por las siguientes industrias farmacéuticas: ALK, AstraZeneca, Chiesi, GSK, Novartis, Teva y Zambón.

Javier Domínguez Ortega declara haber recibido honorarios por su participación como asesor o conferenciante para ALK, AstraZeneca, Bial, Chiesi, GSK, Leti Pharma, Menarini, Mundipharma, Novartis, Sanofi y TEVA.

Jordi Giner Donaire declara haber recibido honorarios por su participación en reuniones, asesorías, o trabajos de investigación organizados por las siguientes industrias farmacéuticas: AstraZeneca, Boehringer Ingelheim, GSK, Mundipharma, Menarini y Pfizer.

Jesús Molina Paris ha recibido honorarios por elaboración de documentos, comité de expertos e impartir conferencias por parte de AstraZeneca, Boehringer, GSK, Menarini, Novartis, Orion Pharma, Pfizer, semFYC, SOMAMFYC y SERMAS.

Navidad Sánchez Marcos declara haber recibido honorarios por su participación en reuniones, asesorías o congresos por parte de GSK, Pfizer, Sanofi, Teva y Zambón.

Vicente Plaza declara haber recibido en los 3 últimos años honorarios por participar como orador en reuniones patrocinadas por AstraZeneca, Chiesi y GSK; y como consultor de ALK, AstraZeneca, Boehringer, GSK y Sanofi. Recibió ayudas económicas para la asistencia a congresos por parte de Chiesi, además de subvenciones para proyectos de investigación provenientes de AstraZeneca, Chiesi y Menarini.
